# Editorial: Omics and Systems Approaches to Study the Biology and Applications of Lactic Acid Bacteria

**DOI:** 10.3389/fmicb.2020.01786

**Published:** 2020-08-20

**Authors:** Konstantinos Papadimitriou, Kimberly Kline, Pierre Renault, Jan Kok

**Affiliations:** ^1^Department of Food Science and Technology, University of Peloponnese, Kalamata, Greece; ^2^Singapore Centre on Environmental Life Sciences Engineering, School of Biological Sciences, Nanyang Technological University, Singapore, Singapore; ^3^Micalis Institute, INRAE, AgroParisTech, Université Paris-Saclay, Jouy-en-Josas, France; ^4^Department of Molecular Genetics, Groningen Biomolecular Sciences and Biotechnology Institute, University of Groningen, Groningen, Netherlands

**Keywords:** genomics, transcriptomics, proteomics, metagenomics, metabolomics, meta-omics, evolution, niche

Early definitions classified lactic acid bacteria (LAB) as Gram-positive, non-sporulating, microaerophilic but aerotolerant, catalase, and oxidase negative bacteria that produce lactic acid. LAB were mostly related to foods as starters, non-starters (NSLAB) and less frequently as spoilers. These definitions necessitated some common evolutionary traits but still remained rather technical and beyond a true evolutionary perspective. For example, diverse bacteria were considered LAB, like members of *Bifidobacteriaceae* and *Lactobacillaceae* families, the first belonging to the Actinobacteria and the second to the evolutionarily distant Firmicutes.

The initial technical definitions of LAB also distort our understanding of the ecological niches they occupy and their functionality with all concomitant negative implications. There are many articles in which LAB are presented to be solely found in the food environment. The use of LAB to produce the vast majority of fermented foods around the world and their unproblematic consumption have led to the notion that LAB are safe *sensu lato*. Furthermore, many LAB are commensals to humans and have probiotic properties. Based on these facts several studies have inaccurately suggested that all LAB are probiotics.

The problems deriving from a technical definition of LAB were always apparent, but systematically neglected. Researchers in the field are familiar with the description of LAB genera related to food. These traditionally included *Lactobacillus, Lactococcus, Streptococcus, Enterococcus, Pediococcus, Leuconostoc, Oenococcus, Tetragenococcus, Carnobacterium*, and *Weissella*. However, even a superficial examination would reveal that not all of these genera are truly food related. In the *Streptococcus* genus, which contains more than 70 species, there is currently only one species acknowledged to be food related, i.e., *Streptococcus thermophilus*. The majority of streptococci are commensals including notorious opportunistic pathogens leading to the death of many humans worldwide every year, like *Streptococcus pyogenes* (Group A *Streptococcus*, GAS), *Streptococcus agalactiae* (Group B *Streptococcus*, GBS), and *Streptococcus pneumoniae*. Thus, it is at least a mishap to characterize the *Streptococcus* genus as food related. A detailed view reveals that important pathogenic bacteria may be present in the LAB genera and have been associated with many different diseases in humans and animals. It should be emphasized that some LAB species are opportunistic pathogens yet found in food, as is the case of *Enterococcus faecalis*, which is a common member of the microbiota of traditional dairy fermented foods while it can cause some of the most serious nosocomial infections.

One way to address these discrepancies is to adopt a definition of LAB based on an evolutionary perspective. Such an approach has been attempted in a number of studies, but their review is beyond the scope of this editorial. Nevertheless, we would like to point out the first chapter of the latest edition of the book entitled “Lactic Acid Bacteria: Biodiversity and Taxonomy” edited by Holzapfel and Wood (2014) where LAB are defined as those bacteria that comprise the order *Lactobacillales* within the class *Bacilli* of the phylum Firmicutes. The order *Lactobacillales* currently includes six families, i.e., *Aerococcaceae, Carnobacteriaceae, Enterococcaceae, Lactobacillaceae, Leuconostoccaceae, Streptococcaceae* with 40 genera and a continually increasing number of species (>400). Other bacteria, which are phylogenetically diverse from Firmicutes or the order *Lactobacillales* but may have common features with LAB (mostly the ability to produce lactic acid), are called “LAB-related.” Such bacteria include members of the *Bifidobacterium, Bacillus*, and *Sporolactobacillus* genera. This strict evolutionary definition may have a major impact on the way we perceive LAB since it circumvents any technical criterion that was used to delineate this group of bacteria. Firstly, LAB as sole members of *Lactobacillales* acquire a “true” phylogenetic relationship beyond that which derives mostly from the ecological niche that they occupy. For example, it should come as no surprise that the dairy *S. thermophilus* is phylogenetically closer to pathogenic streptococci than to *Lactococcus lactis*, which is also mostly a dairy species. Secondly, we may better understand the biology of an LAB member in relation to the entire group. Again, *S. thermophilus* may have traits that are conserved in pathogenic streptococci, yet differences between the two may suggest how the former became avirulent and adapted to the food environment or how the latter inflict virulence to the host. Thirdly, we will be able to objectively address the ecological distribution of LAB. The economic importance and health relevance of starter, probiotic and commensal/pathogenic LAB have attracted most attention up to now. However, we should not overlook that LAB are major food spoilers or that they are extensively associated with plants and plant material and that they have been isolated from unexpected niches, such as soil, marine, and fresh waters, dust, air, etc. With this Research Topic, we would like to raise the awareness of the importance of addressing the shortcomings of technical definitions of LAB while presenting some of the latest developments of LAB research in the era of omics and systems biology.

Over the past three decades, molecular and genetic analysis of LAB species provided important insights into the biology and application of starter and probiotic LAB and in the virulence of LAB pathogens. The knowledge obtained prepared LAB researchers for the forthcoming opportunities provided by the advent of microbial genomics. Today, developments in next-generation sequencing technologies have rocketed LAB genome research and the sequences of several thousands of strains are available. This flood of information has revolutionized our view of LAB. First of all, a detailed picture has emerged about the evolutionary mechanisms allowing LAB to inhabit the diverse ecological niches in which they can be found. Adaptation of LAB to nutrient-rich environments has led in many instances to degenerative evolution processes that resulted in the reduction of the size of their chromosome and the simplification of their metabolic potential. On the other hand, gene acquisition through horizontal transfer is also important in shaping LAB gene pools. Horizontally acquired genes have been shown to underpin niche specialization and technological, probiotic or virulence properties. Progress in bioinformatics tools has allowed rapid annotation of LAB genomes and the direct assignment of genetic traits among species/strains through comparative genomics. In this way, the molecular basis of many important traits of LAB has been elucidated, including aspects of sugar fermentation, flavor formation, production of textural substances, stress responses, colonization of and survival in the host, cell-to-cell interactions, and pathogenicity. Functional genomics and proteomics have been employed in a number of instances to support *in silico* predictions. Given that the costs of advanced sequencing methodologies like RNA-seq are dropping fast, bottlenecks in the *in silico* characterization of LAB genomes will be rapidly surpassed by experimentally verified functional data.

Another crucial advancement in LAB research is the application of systems biology approaches, by which the properties and interactions of components or parts of LAB are investigated to accurately understand or predict their behavior. Practically, systems biology involves the mathematical modeling of complex biological systems that can be refined iteratively with wet-lab experiments. High-throughput experimentation, generating huge amounts of data on the properties and quantities of many components, such as transcripts, enzymes and metabolites, has resulted in several system models of LAB. Novel techniques allow modeling of additional levels of complexity including the function of small RNAs, structural features of nucleic acid molecules, and post-translational modifications. In addition, researchers have started applying systems approaches on LAB multispecies ecosystems in which each species or strain is considered as a part of the system. Metagenomics, metatransciptomics, metaproteomics, and metametabolomics offer the means to combine cellular behavior with population dynamics in microbial consortia.

In this Research Topic we have collected five different review articles. One of the reviews concerns the distribution and functionality of LAB in distinct ecological niches including recent metagenomics evidence for their presence in the human and animal microbiome. George et al. highlight many of the aspects discussed above, including the wide presence of LAB in natural ecosystems beyond the traditional ones. The authors clearly support that in some sites of the human microbiome (e.g., the small intestine or the colon) LAB are certainly subdominant and they exhibit relatively low abundances. This fact may contradict the commonly accepted hypothesis that LAB are of major importance for the human gut microbiome. Taking a step forward, the authors discuss that the presence of LAB should not be considered as advantageous for the host at all times.

The next two review articles focus on how developments in omics technologies are already being employed to study LAB and describe novel applications yet to come in the field. More specifically, in the first review by O'Donnell et al. the authors present a detailed overview of omics methodologies, which have shaped our knowledge in the context of LAB. The need to integrate data from multi-omics experiments to assist systems level understanding of LAB is clearly described. Additionally, the problems arising from integrating and interpreting the multifaceted data produced by multi-omics approaches are also addressed and specific strategies to by-pass such difficulties are proposed. The second review by Liu et al. concerns the application of systems biology, which may lead to improved LAB strains acting as cell factories for the production of food additives and therapeutics, but also as starter cultures. The authors highlight the application of adaptive laboratory evolution (ALE). It is a non-GMO strategy that may result to industrially important phenotypes taking advantage of the inherent genetic variation in a bacterial population exposed to appropriately selected conditions. Mutants deriving from such methods are not the result of single gene manipulations but rather of complex genomic adaptations. In contrast, mutants obtained through metabolic engineering at the systems level are rationally designed by approaches, such as optimization of an enzyme/pathway activity, synthetic biology and *in silico* simulations. Beyond their application as cell factories or improved starters, ALE mutants or mutants produced by systems metabolic engineering can be studied by multi-omics approaches to reveal/verify the mechanisms underlying the observed phenotypes.

The final two reviews describe the most important developments in genomics of specific streptococcal pathogens. Chen reviews how genomics was employed to tackle questions related to virulence mechanisms and epidemiology of *S. agalactiae*, a GBS. As the author underlines, *S. agalactiae* holds an important milestone in bacterial genomics since it was the first bacterium for which a pangenome of eight strains was analyzed. This allowed identifying conserved and variable regions in the genomes. Variable regions could be related to virulence, host and disease specification. Today, more than 7,000 GBS strains have been sequenced, providing a wealth of information concerning the molecular epidemiology of human, cattle and fish isolates. Chen also presents cases of emerging GBS diseases including data suggesting that a foodborne route exists for the transmission of these pathogens, as in the 2015 outbreak in Singapore, which was associated with the consumption of contaminated raw fish. In the final review, Hiller and Sá-Leão focus on the genomic diversity and plasticity of *S. pneumoniae* (pneumococcus). The bacterium is under strong selective pressure from antibiotics and pneumococcal multivalent conjugate vaccines, nonetheless remains a major human pathogen. *S. pneumoniae* colonizes sites of the upper respiratory tract and middle ear in the form of multi-strain biofilms. Studies of the pneumococcal pangenome suggest that more than 20% of the genes in any strain belongs to the accessory genome and, thus, an important number of genes is differentially distributed across *S. pneumoniae* strains. This gene pool may be transferred among pneumococcal strains in the multi-species biofilms through genetic exchange, aiding their prevalence over competitors, their survival of the host immune system and their resistance to antibiotics and vaccines. The authors discuss the striking ability of *S. pneumoniae* of genetic exchange and its implications for the pneumococcal pangenome and biology.

In this Research Topic, we also collected 12 research articles and one method article. Seven of them largely concern the genomics of LAB species. Wels et al. employ comparative genomics in combination with machine learning to resolve the disparity in *Lactococcus lactis* of strains exhibiting a *cremoris* genotype while displaying a *lactis* phenotype. The authors manage to identify orthologous groups of protein sequences that can be used to predict either the taxonomic position or the source of isolation of *L. lactis* strains. In another study, Kelleher et al. define the plasmidome of *L. lactis*. The authors proceed with comparative analysis of newly sequenced and publically available plasmids to identify technologically important traits they may carry. Alexandraki et al. performed comparative genomics of several fully sequenced genomes of *S. thermophilus* to reveal aspects of the evolution, biology and technological properties of the species. The study provides evidence for the existence of lineages within *S. thermophilus* based on the distribution of several genomic traits among its strains. Fontana et al. analyzed the phenotypic and genomic traits of six newly sequenced *Lactobacillus helveticus* strains. Pangenome analysis among many *Lb. helveticus* strains led to identification of specific genes (e.g., genes involved in folate biosynthesis and maltose-degradation as well as multiple copies of 6-phospho-β-glucosidase genes) that may support adaptation to the gut environment and a probiotic potential for some of the new strains. Kazou et al. present the first comprehensive comparative genomics study of *Lactobacillus acidipiscis* isolated from traditional Greek Kopanisti cheese. *Lactobacillus acidipiscis* does not seem to own probiotic genomic traits similar to those found in the phylogenetically relevant *Lactobacillus salivarius*, but it carries pathways for the production of major volatile compounds during the catabolism of amino acids, which may contribute to cheese flavor. Leong et al. performed whole-genome analyses on more than three hundred vancomycin-resistant *Enterococcus faecium* (VREfm) isolates from Tasmania's hospitals. They applied different *in silico* multi-locus sequence typing (MLST) techniques and single-nucleotide polymorphic (SNP) analysis of the isolated strains and determined the relatively rapid spread of a specific sequence type (ST_1421_) to multiple hospitals in an Australian state. Finally, Ayala et al. present some potential probiotic strains with antimicrobial activity against foodborne pathogens. The strains were characterized by typical wet experiments and were sequenced to uncover relevant genes by *in silico* methods.

The Research Topic also includes functional studies of LAB based on transcriptomics or proteomics data. van der Meulen et al. investigate the early transcriptome response of *L. lactis* to environmental stresses using RNA sequencing. Data presented suggests the involvement of many novel previously uncharacterized stress-related genes and small regulatory RNAs that warrants further investigation of how these genomic traits contribute to stress resistance under technologically relevant conditions. Prechtl et al. performed a label-free, quantitative proteomics approach to determine the sucrose-induced response of *Lactobacillus sakei* in relation to dextran formation. Proteomics data in combination with comparative genomics suggest that *Lb. sakei* typically isolated from fermented meat products also exhibits adaptation to plant environments and may have a role as a starter in sucrose-based fermented foods. Spangler et al. test the hypothesis that *Lactobacillus plantarum* may respond to the quorum sensing molecule N-(3-oxododecanoyl)-L-homoserine lactone (3OC12) produced by *Pseudomonas aeruginosa*. Transcriptomics as well as proteomics data indicated that *Lb. plantarum* could sense and respond to 3OC12 by up-regulating genes and proteins of which some were related to its native quorum sensing system.

Finally, three studies have to do with metagenomics. Melkonian et al. present a computational approach that allows identifying properties that distinguish an LAB species within a microbial community using genomic and metagenomic information as well as gene annotation. The authors demonstrate the applicability of their approach by relating the dominance of *Lb. kefiranofaciens* in kefir and the thriving of *Lb. plantarum* in wine fermentation to specific genomic traits of the two species. Seol et al. present a methodological paper that attempts to tackle the issue of the precise determination of probiotic species in food products and probiotic supplements. The authors propose a new sequence coverage-based pipeline that relies on a reference database and can precisely determine probiotic species in products, independently of the sequencing platform used. Last, Gupta et al. use 16S rDNA metagenomics to assess alterations on the distal intestinal microbial communities of Atlantic salmon (*Salmo salar*) during administration of two different probiotic LAB. Each LAB changed the microbial composition of the community, resulting in distinct co-occurrence networks not only against the control salmon population but also between them.

The studies presented in this Topic Editorial represent major contributions made by a wide range of authors from different present-day technological and scientific approaches on the various and contrasted facets through which LABs interfere with food and human health.

## Author Contributions

All authors listed have made a substantial, direct and intellectual contribution to the work, and approved it for publication.

## Conflict of Interest

The authors declare that the research was conducted in the absence of any commercial or financial relationships that could be construed as a potential conflict of interest.
